# Unique Roles of Sphingolipids in Selected Malignant and Nonmalignant Lesions of Female Reproductive System

**DOI:** 10.1155/2019/4376583

**Published:** 2019-05-02

**Authors:** Paweł Knapp, Karolina Chomicz, Magdalena Świderska, Adrian Chabowski, Robert Jach

**Affiliations:** ^1^Department of Gynecology and Gynecologic Oncology, Medical University of Bialystok, Poland; ^2^Ist Medical Faculty with Stomatology, Medical University of Lublin, Poland; ^3^Department of Physiology, Medical University of Bialystok, Poland; ^4^Jagiellonian University Medical College Gynecology and Obstetrics, Krakow, Poland

## Abstract

Cancer develops as a result of the loss of self-control mechanisms by a cell; it gains the ability to induce angiogenesis, becomes immortal and resistant to cell death, stops responding to growth suppressor signals, and becomes capable of invasion and metastasis. Sphingolipids—a family of membrane lipids—are known to play important roles in the regulation of cell proliferation, the response to chemotherapeutic agents, and/or prevention of cancer. Despite the underlying functions of sphingolipids in cancer biology, their metabolism in different malignant tumors is poorly investigated. Some studies showed marked differences in ceramide content between the tumor and the respective healthy tissue. Interestingly, the level of this sphingolipid could be either low or elevated, suggesting that the alterations in ceramide metabolism in cancer tissue may depend on the biology of the tumor. These processes are indeed related to the type of cancer, its stage, and histology status. In this paper we present the unique roles of bioactive sphingolipid derivative in selected gynecologic malignant and nonmalignant lesions.

## 1. Introduction

The developmental profiles of cancer in women can be highly variable. Malignant and nonmalignant lesions of female reproductive system may differ significantly in terms of the degree or type of loss of the self-control mechanisms that accompanies their development and progression.

Age and lifestyle strongly influence the metabolic pathways: angiogenesis, immortality, response to growth suppressor signals, and the capability of invasion and metastasis [1]. In addition, the diagnosis of nonmalignant and malignant lesions in women is not obvious. Therefore, it is important to find new markers that could differentiate premalignant neoplastic lesions from the malignant ones, as well as enabling the monitoring of the course of treatment [2, 3].

Sphingolipids serve as an important function in carcinogenesis. Bioactive sphingolipids, such as ceramide (CER), glucosylceramide (GlcCer), sphingosine (SPH), and sphingosine-1-phosphate (S1P), act as bioeffector molecules and coordinate various aspects of cancer biology, including apoptosis, cell proliferation, cell migration, senescence, and inflammation. In turn, these sphingolipid-regulated processes are crucial in cancer development, progression, metastasis, and chemoresistance of different type of cancer. Moreover, some of them can be used as potential biomarkers for early detection of neoplastic tumors, e.g., ovarian cancer, thus improving the total survival of patients suffering from this type of gynecological malignancies [4]. Sphingolipids are involved in the apoptosis and angiogenesis occurring in the course of neoplastic changes. For instance, S1P participates in the angiogenesis and the process of apoptosis in the ovaries treated with chemotherapy [5–7]. Various analytic methods have been used in order to determine the sphingolipids content, among other things; Thin Layer Chromatography (TLC) followed by Gas Chromatography (GLC) or High Performance Liquid Chromatography (HPLC) are used, often combined with Mass Spectrometry (MS/MS). So far, LC-MS/MS analysis is the most desired method that ensures high sensitivity and structural specificity of the assessment of sphingolipids in small samples of biological material [8].

## 2. Sphingolipid Metabolic Pathways

**Figure 1:**
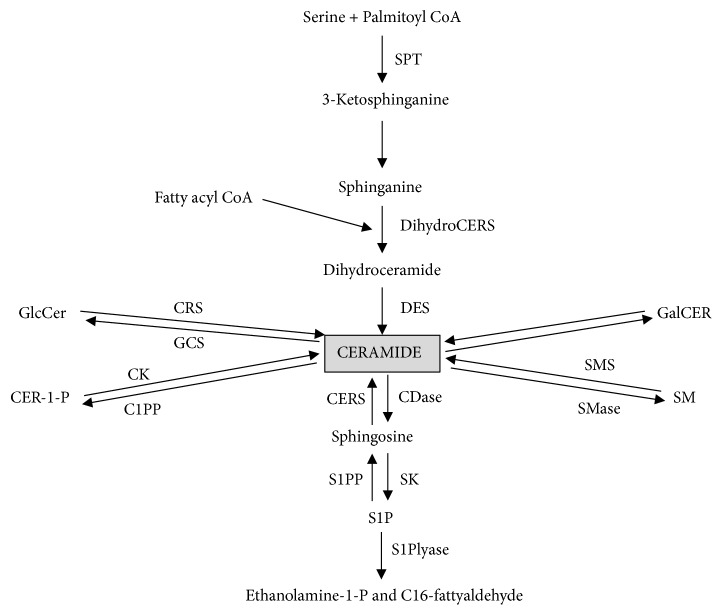
Metabolism of sphingolipids: serine palmitoyl transferase (SPT), (dihydro)ceramide synthase(CERS), dihydroceramide desaturase (DES), diacylglycerol (DAG), sphingomyelinases (SMase), SM synthase (SMS), glucosylceramide (GlcCER), glucosylceramide synthase (GCS), ceramide-1-phosphate (Cer-1-P), ceramide kinase (CK), ceramidases (CDases), sphingosine kinase (SK), cerebrosidase (CRS), and phosphatases of CER-1-P and S1P (C1PP and S1PP).

Ceramides are one of the major lipid molecules of the sphingolipid metabolism and they play a distinctive role in the neoplastic tumor development. They can be generated through the enzymatic hydrolysis of sphingomyelin by sphingomyelinase. Alternatively, CER can be synthesized* de novo* from serine and palmitoyl-CoA (Figure 1) [5]. This is the first but at the same time the rate-limiting stage catalyzed by the enzyme serine palmitoyltransferase. As a result, 3-ketosphinganine is formed, which is directly converted into sphinganine (SPA). Subsequently, the product is acylated by ceramide synthase to dihydroceramide and the latter is converted to ceramide by dihydroceramide desaturase. Ceramide degradation is mainly caused by deacylation to SPH by the enzyme ceramidase. On the other hand, SPH may be reacylated to CER or transformed into sphingosine-1-phosphate by sphingosine kinase (SPHK) [5, 9]. What is important, S1P may be involved in breast cancer, endometrial cancer, and ovarian cancer progression [6, 10, 11].

Recent research has revealed that ceramides constitute an essential antineoplastic factor, since those molecules can act like lipid tumor suppressors. Their proapoptotic as well as growth-inhibiting properties have gained much attention in the field of gynecological oncology. Not only CER, but also the elevated levels of SPH and SPA may influence the tumor suppressing processes like apoptosis or differentiation. Several studies have demonstrated that the incubation of tumor cell lines with CER analogs causing ceramide accretion is in line with caspase activation resulting in the apoptosis of cancer cells [3, 12–16].

**Figure 2:**
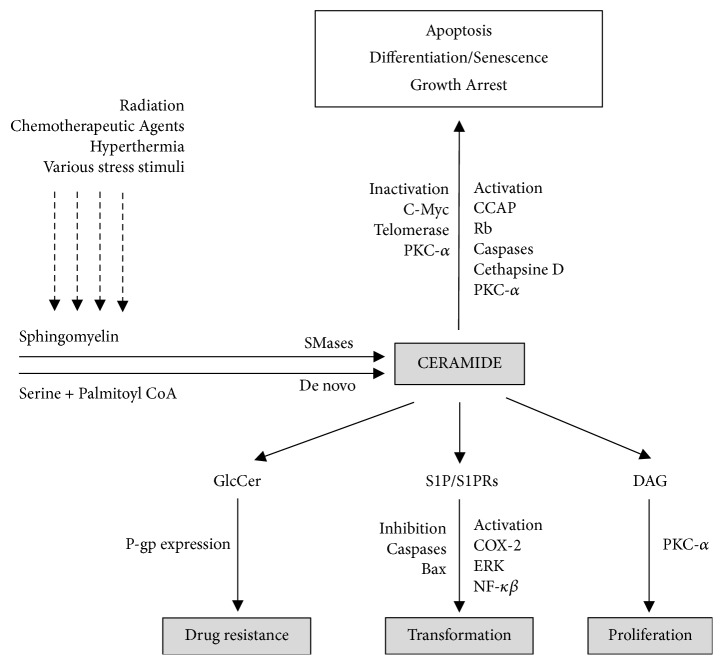
The biological mechanism of sphingolipids involved in the regulation of cancer growth and therapy: ceramide-activated protein phosphatases (CAPP) and diacylglycerol (DAG).

In contrast, S1P is regarded as a tumor-promoting compound involved in cell proliferation, transformation, inflammation, and angiogenesis. Moreover, an increase in the expression of sphingosine kinase (SPHK1), an enzyme that transforms sphingosine into S1P, causes the accumulation of S1P in the cell, thus promoting carcinogenesis and tumor formation. S1P, apart from being a messenger in cell signaling, also binds to membrane receptors coupled to a G protein (S1PRs), which is responsible for the majority of its biological actions (Figure 2) [5]. There is strong likelihood that large amounts of S1P produced by the neoplastic tissue stimulate the ovarian cancer cell proliferation by binding with the above-mentioned receptor [1, 3, 4]. The dysregulation of sphingolipid metabolism, such as the overproduction of ceramides and their conversion to S1P, can trigger neoplasm development. The imbalance on the CER/S1P axis resulting in the excessive production of S1P may contribute to the increased risk of ovarian cancer. Alberg et al. drew an identical conclusion and reported that the upregulation of bioactive sphingolipids in lung cancer can also potentially be due to the dysregulation of S1P metabolism [17]. A rise in the content of S1P is also observed in endometrial carcinoma. This is consistent with the activation of SPHK1 and the accumulation of sphingosine. It is becoming evident that the S1P/SPHK1 metabolism participates in the main oncogenic mechanisms, from cell survival or proliferation to the apoptosis prevention. Interestingly, the S1PRs mentioned above may take effect in both auto and paracrine manner, since the extracellular level of S1P has been observed. Accordingly, an elevated level of S1P in neoplasm tissue does not precisely follow the range of S1P/SPHK1 pathway stimulation in endometrial carcinoma. Such data becomes more accessible, as the concentration of S1P in matrix was higher in endometrial cancer patients, because of its greater production and export by the neoplastic tissue [11, 18]. A similar remark was made by Sutphen et al. regarding subjects with ovarian cancer [19]. On the contrary, a low serum level of S1P observed in ovarian cancer patients results from its consumption by biologically aggressive tumor cells that constantly proliferate [6]. Additionally, the disrupted metabolism of CER and SP1 may be responsible for the resistance to chemotherapy, which is also caused by the presence of protein mediators and their signaling actions. As reported by Gatt et al., the elevated expression of ceramide-transporting proteins and their intracellular accumulation coupled with a high level of S1P is a potential cause of resistance to chemotherapy with platinum derivatives in ovarian cancer [20].

Recent research has revealed considerable dissimilarities in CER content between various cancers and particular healthy tissue, thereby indicating that changes in ceramide metabolism in neoplasm may depend on the type of tumor [1, 3, 9, 21]. Our previous study demonstrated that in uterine leiomyomas the content of basic sphingolipids remains quite constant. However, the increase in CER level with a concomitant decrease in S1P concentration was observed as compared to normal uterus [22]. Based on our previous research we may also speculate that the neoplastic progression towards more aggressive neoplasms is related to the marked increase in sphingolipid content. The dysregulations in sphingolipid metabolism were also observed in malignant lesions of the female reproductive system, such as endometrial and ovarian cancer [6, 11]. This claim is supported by the fact that the content of all measured sphingolipids, ceramide species, and enzymes catalyzing the transformation to bioactive derivatives was upregulated in the neoplasm tissue. Additionally, the accumulation of sphinganine and dihydroceramide coupled with the activation of palmitoyltransferase suggests that ceramides are synthesized* de novo *[12, 23–25]. This remains in agreement with other reports that present a remarkably elevated content of dihydroceramide and an increased level of SPT in several types of carcinoma. However, the* de novo* CER synthesis pathway is not characteristic or obligatory for all tumors. This thesis has been proposed in the publication of Carton et al. who demonstrated, with the use of immunohistochemistry, that only half of 18 various malignant tissues expressed elevated SPT subunits [26].

Interestingly, depending on the lengths of lipid chains, ceramides can play opposing roles in neoplastic tumors. The analysis of C18-Cer function demonstrated its role in tumor suppression, while C16-Cer was shown to induce cell proliferation in ovarian cancer. This indicates how many factors influence various CER metabolism actions [1, 27–29].

High concentration of total ceramide in ovarian cancer as well as the one we can observe in endometrial carcinoma seems puzzling in the context of its proapoptotic properties [9]. The first reasonable explanation is that the activation of the CER pathway fulfills the demand of proliferating tumor cells, since ceramides are crucial components of the biological membranes. Another possible interpretation regarding the endometrial carcinoma is probably related to the overactivation of ceramidase, which is supported by the accumulation of sphingosine [11]. Likewise, in ovarian cancer it is due to the increased rate of degradation by the same enzyme [6]. Park et al. described this mechanism by presenting increased activation of acidic ceramidase in many cancer patients [30]. Moreover, those growth-arresting features of CER could be antagonized by more intensive production of S1P as the ratio S1P/ceramide rather than the presence of each separate sphingolipid determines a cell's fate. In endometrial cancer this ratio remained the same as in the healthy uterine. Finally, such paradox could also be explained by the fact that neoplastic cells might develop resistance to ceramide [11].

## 3. Sphingolipids as Diagnostic Biomarkers

Plasma concentration of selected sphingolipids could be an essential marker in ovarian cancer diagnostics. New biomarkers for noninvasive testing are urgently needed because of the shortage of highly specific and sensitive methods for detecting early ovarian cancer, in particular in respect of the serous ovarian cancer (SOC), one of the most common types of ovarian neoplasm, which lacks specific clinical manifestations at an early stage [31, 32]. Diverse evidence shows the involvement of sphingolipids in different pathways associated with their elevated levels (e.g., preterm labor, preeclampsia, fetal Down syndrome, and myocardial infarction). Murphy et al. demonstrated the reduced content of sphingolipids in the brain tissue of people with Down syndrome. This is associated with the delayed myelination of neurons in the developing brain of children with trisomy of chromosome 21, resulting in their mental retardation [32].

Interestingly, other studies showed that in many human diseases the metabolism of unique sphingolipids is affected in plasma, as well as in erythrocytes and platelets [33–35]. Knapp et al. demonstrated that patients with acute myocardial infarction are characterized by a reduction in plasma S1P and sphinganine-1-phosphate (SA1P) concentrations that is maintained for as long as 30 days after the infarction, with only partial recovery two years later [36]. In addition to plasma, S1P and SA1P are present in high concentrations also in the blood cells. Until recently, it was thought that platelets are the main source of plasma sphingoid base-1-phosphates. Thrombocytes possess highly active SPHK and do not express S1P lyase activity, which allows them to accumulate S1P. However, it was demonstrated that human platelets release S1P only upon activation. Similarly to platelets, thrombocytes are able to incorporate extracellular sphingosine and convert it to S1P. In addition, erythrocytes do not show measurable activity of S1P-degrading enzymes. However, in contrast to thrombocytes, red blood cells secrete S1P and SA1P spontaneously. Knapp et al. showed that patients with acute myocardial infarction were characterized by a striking accumulation of both S1P and SA1P in erythrocytes. A similar, albeit transient, and less prominent trend was observed in platelets as well [36–38].

Therefore, the purpose of our previous research was to analyze how the levels of specific sphingolipids (ceramides, sphingosine-1-phosphate, sphingosine, and sphinganine) are changed in serum, red blood cells, and platelets of patients diagnosed with advanced serous ovarian cancer (ASOC) [39]. Our observations of a notably higher plasma level of C16-Cer, C18:1-Cer, and C18-Cer in the advanced ovarian cancer group were compared with the results of the control one. Similarly, we noticed a considerably higher concentration of C18:1-Cer in the erythrocytes in the same examined neoplasm tissue in contrast to the control group. To establish the diagnostic potential of the sphingolipids, all significant measurements were later used in ROC curves. Through the setting of the threshold values, the prediction of the likelihood of ASOC was made with specific sensitivity and specificity. ROC analyses allow the differentiation of patients with moderate/severe vs. mild ovarian cancer. We demonstrated a significantly increased risk of ovarian cancer, as the plasma concentration of each ceramide was as follows:

C16-Cer > 315.98 ng/100 ml; C18:1-Cer > 4.81 ng/100 ml and C18-Cer > 113.26 ng/100 ml; RBC C18:1-Cer > 0.1 ng/100 ml. Additionally, C16, C18.1, and C18 plasma levels corresponded with the progression of the tumor: the FIGO staging and grading [6, 39].

Interestingly, hardly the individual measurement of the aforementioned CER concentration in plasma differentiates patients and therefore those ceramides may be useful as potential biochemical markers. A unique switch into the carcinogenesis pathways recognized as the elevation of particular CER concentrations across cut points seems helpful in taking further steps in the diagnosis. The estimated levels of plasma C16-Cer, C18:1-Cer, and C18-Cer could be a crucial factor in making the decision regarding an adequate surgery or procedure [6, 39], such as a biopsy, which is frequently performed when the tumor is in the development stage III/IV. Furthermore, our previous research demonstrated different levels of the total investigated CER in RBC, platelets, and plasma of patients with ASOC. The results were as follows: plasma: 4236.4 vs. RBC: 100.1 vs. PLT: 444.1 [ng/100 ml]. The metabolic changes allow us to ascertain that CER are synthesized* de novo*. This is due to the raised concentrations of enzymes catalyzing the conversion to bioactive derivatives, as well as the accumulation of SPA and dihydroceramide in conjunction with the activation of palmitoyltransferase. Based on this data we may conclude that plasma CER mostly originate from neoplastic tissue. The constant levels of selected sphingolipids in the blood cells both in RBC and in platelets may imply the autonomous control of their metabolism. This corresponds to the findings of Blachnio-Zabielska et al. who documented similar stable levels of those sphingolipids in RBC and PLT with a reduction in the levels of CER, SPH, and SPA in contrast to S1P in the liver of rats treated with myriocin [33]. Some of the sphingolipids are unaltered in the blood cells of patients who suffer from ASOC due to the high grade of malignancy of this tumor which produces S1P responsible for cell proliferation, neoangiogenesis, and antiapoptotic processes [6, 39]. Presently, an increasing number of studies are dedicated to markers in the advanced stages of ovarian cancer [40–45]. One of the most important factors in the tumor transformation, invasion, and spreading of cancer cells is angiogenesis. Since there is no data published, we can consider that carcinoma tissue S1P—as a proangiogenic factor—stimulates the receptors found on the endothelial cells located in preexisting capillaries. As a result, the basement membrane is destroyed owing to the S1P and protease release which allows endothelial cells to proliferate into the immediate surroundings. In ovarian cancer, there is probably a tendency for reduction in S1P release from the endothelial cells with a subsequent decrease in the plasma level.

Additionally, the compound of S1P in the described ovarian cancer is decomposed by various unspecific phosphatases, the activity of which may be increased in this type of tumor. At the same time, the aggressive biological nature of the above cancer contributes to the destruction of endothelial cells at the advanced stages of the disease. We may conclude that, because of the malignancy of this neoplasm, most of the described blood cell sphingolipids are used in many processes and despite the upper level of platelets, the total levels of sphingolipids are quite constant [6, 46, 47].

## 4. Sphingolipid Metabolism and Its Impact on Cancer Treatment 

Undoubtedly, the proposed cut-off values for unique ceramides may prove practical, since we can refer patients for chemotherapy or a surgical procedure [6]. The first method of treatment can sometimes be very limited due to a cancer's resistance to chemotherapy [48]. As it has been already mentioned, ovarian cancer can be caused by S1P dysregulation and the signaling actions carried out by protein mediators. On the other hand, in the case of endometrial carcinoma, the upregulation of SPHK1 may result in the resistance to doxorubicin and cisplatin, generally used in the treatment of metastases of this tumor. Other researchers demonstrated that the tumor's resistance not only to chemotherapy, but also to the radiation therapy is a consequence of the increase in the level of SPHK1. As regards the endometrial cancer, there is a high probability that a limited response to chemotherapy is associated with changed sphingolipid metabolism. Curiously, the antineoplastic action of these chemotherapeutics depends to a considerable extent on the rise in plasma ceramide concentration, primarily resulting from the stimulation of the* de novo* synthesis pathway [6]. The activation of this pathway in endometrial cancer was previously characterized. It is worth mentioning that the catabolism of CER is stimulated as SPH is accumulated. We assume that resistance to chemotherapy in endometrial carcinoma, similarly to that observed in ovarian cancer, results from the altered sphingolipid metabolism specified by multiple factors. From the practical point of view, the previous publications concerning the identification of new therapies indicate that decreasing S1P rather than boosting CER concentration needs to be considered in endometrial carcinoma treatment. This approach may contribute to the improvement of the effectiveness of conventional chemotherapeutics [11].

A new therapeutic idea is the suppression of glucosylceramide synthase, which works by attenuating the proapoptotic activity of ceramide in cancer cells. Researchers suggest using Glc as a preclinical support for ovarian cancer therapy [49]. However, the problem results from the analytical methods that do not allow a direct assessment of the role of glucosylceramide synthase in the course of chronic diseases [50].

From a clinical point of view, bioactive sphingolipids may be useful for tumor detection at an early stage as potential biomarkers for ovarian cancer. Sphingolipid metabolism in benign tumors is poorly investigated, as only very few studies present data regarding the CER/S1P axis. Nonetheless, the alterations in CER metabolism play an essential role not only in malignant (ovarian cancer; endometrial cancer), but also nonmalignant lesions, such as uterine leiomyomas. In summary, it must be emphasized that the metabolism of sphingolipids is related to cancer progression. It is important to recognize the crucial role of ceramide in order to differentiate selected malignant and nonmalignant lesions of female reproductive system not only in the pathogenesis of the disease, but also as a potential target of new therapies.
